# Dirac and Weyl Semimetal in *XY*Bi (*X* = Ba, Eu; *Y* = Cu, Ag and Au)

**DOI:** 10.1038/srep14423

**Published:** 2015-09-24

**Authors:** Yongping Du, Bo Wan, Di Wang, Li Sheng, Chun-Gang Duan, Xiangang Wan

**Affiliations:** 1 National Laboratory of Solid State Microstructures and Department of Physics, Nanjing University, Nanjing 210093, China; 2 Collaborative Innovation Center of Advanced Microstructures, Nanjing University, Nanjing 210093, China; 3 Key Laboratory of Polar Materials and Devices, Ministry of Education, East China Normal University, Shanghai 200062, China

## Abstract

Weyl and Dirac semimetals recently stimulate intense research activities due to their novel properties. Combining first-principles calculations and effective model analysis, we predict that nonmagnetic compounds Ba*Y*Bi (*Y* = Au, Ag and Cu) are Dirac semimetals. As for the magnetic compound Eu*Y*Bi, although the time reversal symmetry is broken, their long-range magnetic ordering cannot split the Dirac point into pairs of Weyl points. However, we propose that partially substitute Eu ions by Ba ions will realize the Weyl semimetal.

Following the discovery of topological insulator (TI)^1^,^2^, there has been considerable research interest in studying the Weyl semimetal (WSM), the first metallic topologically nontrivial matter^3^,^4^,^5^,^6^. In WSM, non-degenerate valence and conduction bands touch at an accidental degeneracy point in a three-dimensional (3D) Brillouin zone, and its low energy physics is approximated by the Weyl equation^3^,^4^. Weyl points, the nondegenerate linear touchings of the bulk bands, always come in pair, and they are robust due to the protection by the topology of the band structure. The most remarkable feature of WSM is the Fermi arc surface states^3^. Several compounds, including pyrochlore iridates^3^, TI based heterostructures^7^, HgCr_2_Se_4_^8^ and many other systems^9^,^10^,^11^,^12^,^13^ had been theoretically predicted as promising WSMs. The indication about realization of WSM have been reported^14^,^15^,^16^. Very recently, the noncentrosymmetric and nonmagnetic transition-metal monophosphide are predicted as WSM^17^,^18^ and the Fermi arc, as the smoking-gun feature, has been confirmed experimentally^19^,^20^,^21^,^22^,^23^.

Same as the WSM, the Dirac semimetal (DSM) is also a 3D analog of graphene^24^,^25^,^26^,^27^. But in contrast with Weyl point, the Dirac point has four-fold degeneracy, and does not possess a topological number, consequently the Dirac point is not robust against the external perturbations and usually hard to be realized. Thus the 3D DSM receive much less attention until the discovery of Na_3_Bi^28^ and Cd_3_As_2_^29^. Wang *et al*. find that there is a paired 3D bulk Dirac points exist on the *k*_*z*_ axis of Na_3_Bi^28^ and Cd_3_As_2_^29^, and these Dirac points are protected by the crystal symmetry thus are stable^28^,^29^. The theoretical prediction of DSM in Na_3_Bi and Cd_3_As_2_^28^,^29^ had been quickly confirmed by the subsequent photoemission measurement^30^,^31^,^32^,^33^. This immmediately triggers a new wave of research to explore the unique properties associated with the 3D Dirac points in the DSM^30^,^31^,^32^,^33^,^34^,^35^,^36^. Unfortunately, Na_3_Bi is not stable in air while arsenic limits the application of Cd_3_As_2_. Therefore searching new 3D DSM that is stable in nature and less toxic is of both fundamental and technological importance.

In this paper, based on the density functional theory (DFT) calculations and effective low energy models, we predict that Ba*Y*Bi (*Y* = Au, Ag and Cu) are promising 3D Dirac materials. For BaAuBi, the nontrivial topology is due to the band inversion of the Bi-*p* bonding and antibonding states, while for the BaAgBi and BaCuBi, the band inversion happens between the Ag/Cu *s* and Bi *p* orbital. Protected by the *C*_3_ rotation symmetry, the Dirac points locate along the Γ − *A* line. The magnetic configuration in Eu*Y*Bi indeed break the time reversal symmetry, however cannot split the Dirac point into two Weyl points. We propose that partially substituting Eu by Ba, i.e. alloy compound Eu_*x*_Ba_(1−*x*)_Ag(Au)Bi, which could be grown using molecular beam epitaxy (MBE) technique, is a promising way to realize the WSM.

## Results and Discussion

**Figure 1:**
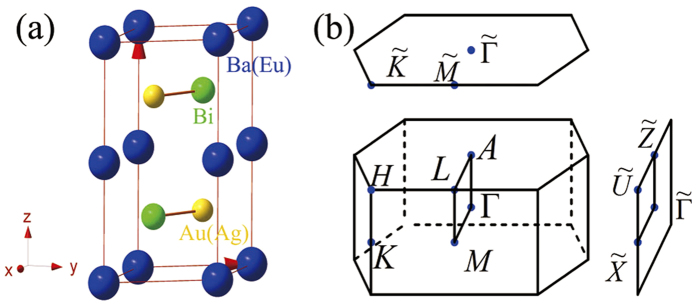
(**a**) Crystal structure of BaAuBi. BaAgBi and BaCuBi have similar structure. (**b**) Brillouin zone of bulk and the projected surface Brillouin zones of (001) and (010) planes.

Ba*Y*Bi (*Y* = Au, Ag, Cu) crystallize in the same hexagonal ZrBeSi type structure with space group 
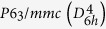
37. The crystal structure of BaAuBi is shown as an example in Fig. 1, in which Au and Bi ions form honeycomb lattice layers stacking along *c* axis and sandwiched by trigonal layers formed by Ba atoms. There are two formula units in the primitive unit cell, and the six atoms in the unit cell locate at three nonequivalent crystallographic sites: Ba atoms occupy the 2*a* 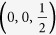, while Au and Bi reside on the 2*c* 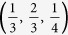 and 2*d* 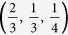 sites respectively^37^. There is no free internal coordinates, and the lattice constants are the only structural parameter for Ba*Y*Bi lattice. We optimize the lattice parameter and for all of the three compounds, our numerical lattice constants are in good agreements with experiments, and the small discrepancy between the numerical and experimental structure has negligible effect on the electronic structure. Hence, the following results are obtained based on the experimental structure, unless stated specifically.

**Figure 2:**
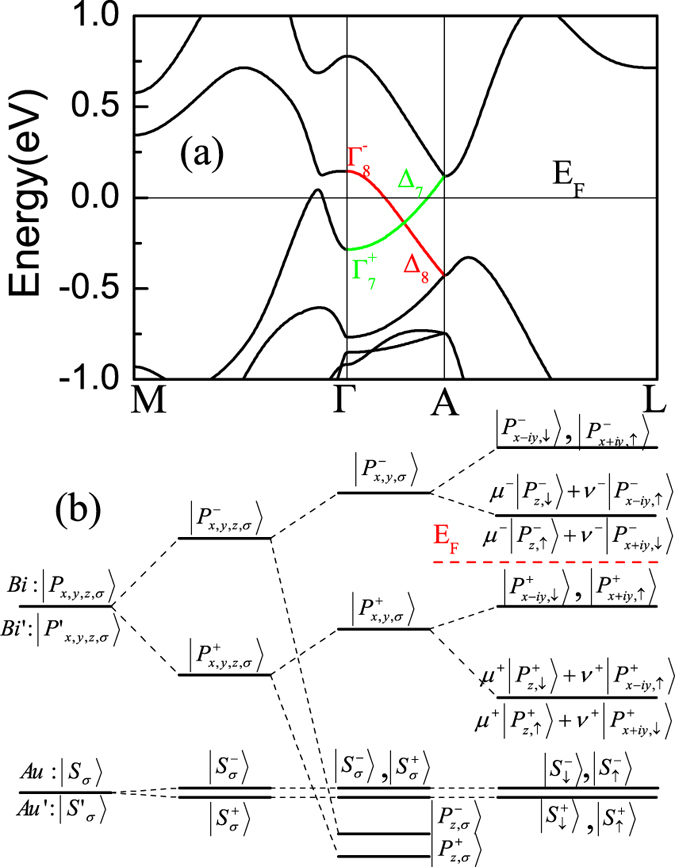
(**a**) Electronic structure of BaAuBi. Green and red line highlights the different irreducible representation along Γ − *A* line. (**b**) Band evolution near Fermi energy of BaAuBi at Γ point, red dashed line stands for the Fermi energy (see main text for detailed description).

We first calculate the electronic structure of BaAuBi, and show the results in Fig. 2(a). The Ba in BaAuBi is highly ionic, has negligible contribution to the states around Fermi level. Au-6*s* and 5*d* bands mainly located at −4 to −1 eV, and −6 to −4 eV, respectively. The Bi-6*s* is basically located about −11 eV below the Fermi level. The valence and conduction bands are dominated by the Bi-6*p* bonding and antibonding states. Checking the wave function, we find that at the Γ point the Bi-6*p* antibonding state is higher than the Bi-6*p* bonding state, however at the *A* point, the odd-parity state is about 0.545 eV lower in energy than even-parity state.

In order to understand the mechanism of the band inversion, we illustrate the band evolution at the Γ point of BaAuBi in Fig. 2(b). As discussed above, the states near Fermi level are primarily contributed by the Bi-6*p* orbital, with also the Au-6*s* state. Since the two Bi atoms (Bi and Bi′) in the unit cell are related to each other by the inversion symmetry, similar with ref. 38,39, we combine the Bi-6*p* orbitals to form the hybridized states and label the bonding and antibonding states as 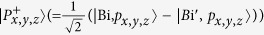 and 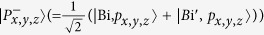 respectively, where the superscripts +/− denote the parity of the corresponding states. According to the point group symmetry, the *p*_*z*_ orbital split from the *p*_*x*_ and *p*_*y*_ orbitals while the latter two are still degenerate as shown in the Fig. 2(b). Finally, we consider the effect of SOC. The 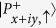 and 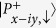 states are pushed up by the SOC, while the 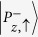 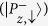 will mix with 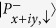 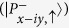, consequently the bonding 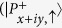 and 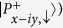 and antibonding states 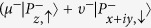 and 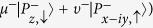 are close to each other at the Γ point, and the band inversion occurs at the *A* point as shown in Fig. 2.

Along Γ − *A* line the C_3_ symmetry is preserved, by the symmetry analysis the two relevant bands along this line belong to different representations (Δ_7_ and Δ_8_ as shown in Fig. 2(a). See Additional Data for the detail). Thus the hybridization between these bands is strictly forbidden, which results in the protected band crossing as shown in Fig. 2(a). The linear band dispersions associated with Dirac points near the Fermi surface will contribute a high-field unsaturated linear magnetoresistance^40^,^41^, we thus believe such novel properties may also be observed in BaAuBi, although the Dirac point is located slightly below the Fermi level as shown in Fig. 2(a).

Since the topological nature is determined by the Δ_7_ and Δ_8_ bands, based on the projection-operator method (see Additional Data), we build the effective Hamiltonian by using the four relevant states as basis vectors (in the order of 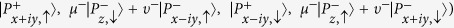 at Γ point. We neglect all of other states, since they are far from the Fermi level and do not involve into the band inversion, and the Hamiltonian can be written as:


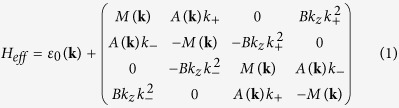


where 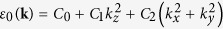, 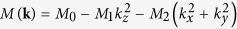, 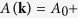 
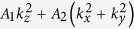
 and *k*_±_ = *k*_*x*_ ± *ik*_*y*_. The parameters in the above formula are material dependent, and by fitting the DFT calculated band dispersion, we obtain *C*_0_ = −0.06978 *eV*, *C*_1_ = −0.34038 *eV* ⋅ *å*^2^, *C*_2_ = 2.25 *eV* ⋅ *å*^2^, *M*_0_ = −0.21537 *eV*, *M*_1_ = −1.9523 *eV* ⋅ *å*^2^, *M*_2_ = −7.9507 *eV* ⋅ *å*^2^, and *A*_0_ = 1.3668 *eV* ⋅ *å*. Solving the above eigenvalue problem, we obtain 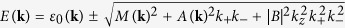, and at 
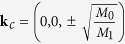
, we get the gapless solutions. In the vicinity of **k**_*c*_ and neglect the high-order terms, *E*(**k′**) would be equal to 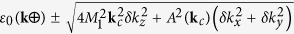 (*δk*_*x*,*y*,*z*_ are small displacement from **k**_*c*_), which is a linear dispersion and suggests in neighbourhood of **k**_*c*_, our effective Hamiltonian is nothing but 3D anisotropic massless Dirac fermions. A 3D Dirac semimetal state can also be realized at the critical point of the topological phase transition between a band insulator and a 3D topological insulator^42^,^43^. Different from this case, the Dirac points in BaAuBi are protected by the C_**3**_ rotation symmetry, thus very robust.

**Figure 3:**
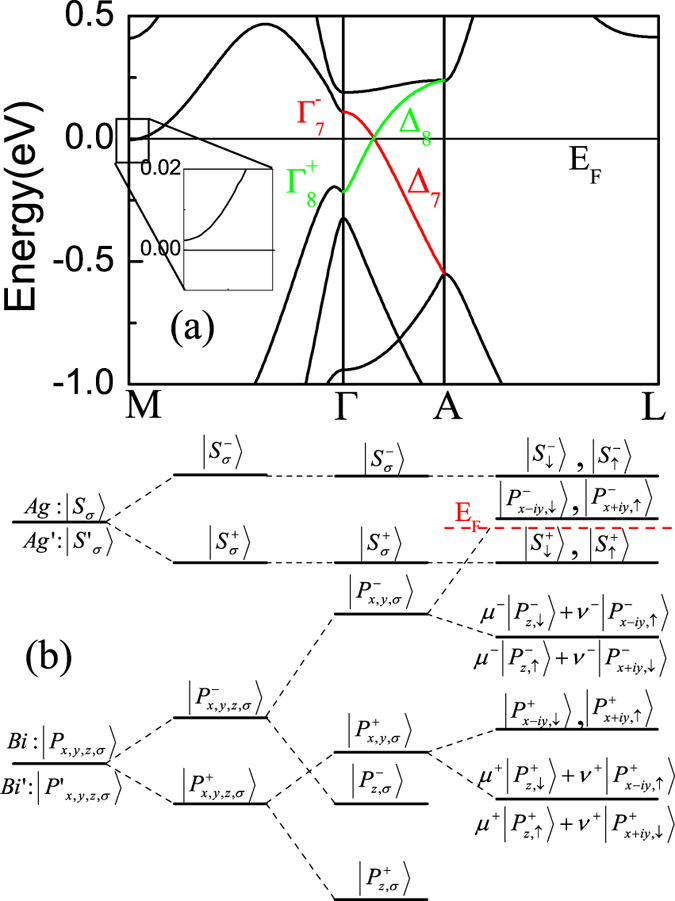
(**a**) Electronic structure of BaAgBi, Green and red line highlights the different irreducible representation along Γ − *A* line. (**b**) Band evolution around Fermi energy of BaAgBi at Γ point, red dashed line stands for the Fermi energy.

We also investigate the BaAgBi and BaCuBi. The electronic properties of BaCuBi are very similar with that of BaAgBi, we thus only report results of BaAgBi. As shown in Fig. 3(b), significantly different with the Au-6*s* state in BaAuBi, the Ag-5*s* orbit in BaAgBi is higher in energy than Bi-6*p* state, consequently the states closed to the Fermi level become 
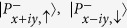
 and 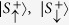. Similar with the case in Na_3_Bi^28^, due to the strong SOC of Bi-6*p*, the 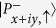 and 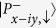 states will be pushed up, which result in the band inversion at Γ point. This inversion is confirmed by our DFT calculation, as shown in Fig. 3(a), at the Γ point, the 
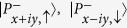
 is higher than 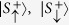 by about 0.34 eV. Along Γ − *A* line, these two bands belongs to different representations (Δ_7_ and Δ_8_), thus there is also a unavoidable crossing point located at Γ − *A* line. It is also easy to prove that the band dispersion is linear around the band touching points. Thus, the crossing points are the Dirac points.

The Dirac points in BaAgBi are doubly degenerate due to inversion and time-reversal symmetries, and upon breaking the time reversal symmetry^3^ or inversion symmetry^42^,^43^ a Dirac cone will split into two Weyl nodes separated in momentum space. This family of intermetallic compound with hexagonal structure indeed has several members with magnetic ion Eu: Eu*XY* (*X* = Cu, Au, Ag; *Y* = As, Sb, Bi)^37^,^44^,^45^. Experiments confirm that some of them indeed possess long-range magnetic configuration^44^. Unfortunately, the Eu^2+^ spins align ferromagnetically with the *ab* plane, but antiferromagnetically along the *c*-axis^45^, therefore the exchange field is exactly cancelled at the *XY*-plane of Eu*XY*. Thus breaking the time reversal symmetry by this type of antiferromagnetic configuration cannot split the Dirac points, and the compounds of Eu*XY* have no chance to become WSM.

**Figure 4:**
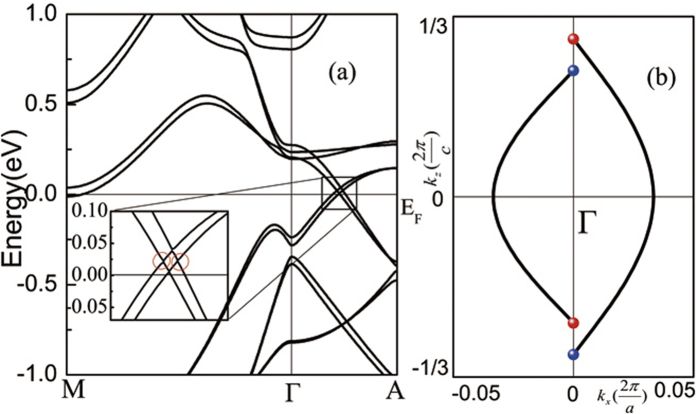
Band structure and surface state of Eu_0.5_Ba_0.5_AgBi. (**a**) Calculated band structure of Eu_0.5_Ba_0.5_AgBi.(**b**)The sketch of the Fermi arcs connecting projected bulk Weyl points of opposite chirality. The blue and red dots denote the Weyl points with opposite chirality.

We, however, expect that substituting part of Eu ions by Ba ions, the two antiferromagnetically coupled Eu plane in Eu_*x*_Ba_1−*x*_AgBi may not exactly cancel each other, and then there is a chance the compound becomes WSM. To confirm this expectation, we then performed another calculation on Eu_0.5_Ba_0.5_AgBi, in which we replace one of the two antiferromagnetically coupled Eu plane in the unit cell by Ba. According to our calculation, the *c* axis is the magnetic easy axis, this magnetization does not break the C_**3**_ rotation symmetry, consequently as shown in Fig. 4(a), the Dirac point indeed splits into two Weyl points as marked by red circle. There is also two other Weyl points slightly above/below the Weyl nodes marked by red circle. The Weyl nodes are very close to the Fermi energy as shown in Fig. 4(a), thus, the phenomena associated with the chiral anomaly^46^,^47^,^48^ also exist in Eu_0.5_Ba_0.5_AgBi. Fig. 4(b) shows the Fermi arcs which connect projected bulk Weyl points of opposite chirality. Thus we believe to grow it by the cutting edge film growth technique like MBE and to explore the possible WSM are a very interesting topic.

In summary, based on density-functional calculation and effective model analysis, we propose that the Ba*Y*Bi (*Y* = Au, Ag and Cu) are 3D Dirac semimetals. The nontrivial topological feature is due to *p-p* inversion for BaAuBi and *s-p* band inversion for BaAgBi and BaCuBi, and their Dirac points are protected by the *C*_3_ rotation symmetry and thus are very robust. Their magnetic cousins, i.e Eu*Y*Bi (*Y* = Au, Ag and Cu) are not Weyl semimetals. However, partial substitution of Eu with Ba ions in Eu*Y*Bi could result in the Weyl semimetal. Furthermore, our numerical calculation also confirm that a uniaxial strain along *a*-axis, which breaks the *C*_3_ rotation symmetry, will drive BaAgBi into topological insulator.

*Note*. When finalizing our work, we became aware of a recent study by Borisenko *et al*.^49^, in which the authors also predict BaAgBi is a possible 3D DSM, agreeing with our conclusion.

## Methods

The electronic band structure calculations have been carried out using the full potential linearized augmented plane wave method as implemented in WIEN2K package^50^. The modified Becke-Johnson exchange potential together with local-density approximation for the correlation potential (MBJLDA) has been used here to obtain accurate band inversion strength and band order^51^. A 16 × 16 × 7 mesh is used for the Brillouin zone integral. Using the second-order variational procedure, we include the spin-orbital coupling (SOC) interaction.

The tight-binding model has been established by using Slater-Koster method^52^. 5 s orbit of Ag atom and three 6p orbits of Bi atom are taken as basis. The tight-binding model has been used to simulate the bulk band structure with Weyl points as found by our LSDA + SO + U calculation. To calculate the surface state and Fermi arc, we build the (010) slab of a thickness of 85 unit-cells.

## Additional Data

### Effective Hamiltonian for BaAuBi

The conduction and valence bands of BaAuBi are mainly contributed by four states: 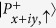, 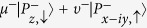, 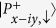 and 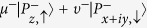, we thus use these states as the basis to build the effective model Hamiltonian at the Γ point of BZ. As a 4 × 4 hermitian matrix, the effective Hamiltonian can be written as 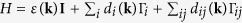, where **I** is the 4 × 4 identity matrix, Γ_*i*_ and Γ_*ij*_ are Dirac matrices, *ε*(**k**), *d*_*i*_(**k**), and *d*_*ij*_(**k**) are function of momentum *k*.

The Hamiltonian should be invariant under the operation of crystal symmtery and time reversal symmtery. This requires the function *d*_*i*_(**k**) [*d*_*ij*_(**k**)] and the associated Γ_*i*_ [Γ_*ij*_] matrices belong to the same irreducible representation. Thus the key problem is to determine the irreducible representation for *d*_*i*_(**k**) [*d*_*ij*_(**k**)] and Γ matrices, which can be done by the projection-operator method.

The Dirac Γ matrices can be written as Γ_1_ = *σ*_1_ ⊗ *τ*_1_, Γ_2_ = *σ*_2_ ⊗ *τ*_1_, Γ_3_ = *σ*_3_ ⊗ *τ*_1_, Γ_4_ = *σ*_0_ ⊗ *τ*_2_, Γ_5_ = *σ*_0_ ⊗ *τ*_3_, and Γ_*ab*_ = [Γ_*a*_, Γ_*b*_]/2*i*^39^. The projection operator is defined as 
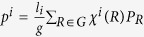
, where *g* is the group order, *l*_*i*_ is the dimension of the *i*th representation, *R* denotes the group element i.e. the symmetry operation, *χ*^*i*^(*R*) represent the character of group element *R* in *i*th representation, *P*_*R*_ is the operator of group element *R*.

The double group of 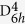 has 18 classes, and their irreducible representations are denoted as *R*_1_ to *R*_18_^53^, and its character table can be found in ref. 45. Based on the basis mentioned above, one can easily work out the transformation matrix *D*_*R*_ for symmetry operator *P*_*R*_, which allow us to apply the projection operator *p*^*i*^ on 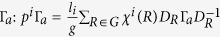, consequently determine the irreducible representation of Γ_*a*_. Using the same process, one can also determine the irreducible representation for the polynomials of **k** up to *O*(**k**^3^). We present the irreducible representation of Dirac Γ matrices and polynomials of **k**, and their transformation under time reversal in Table 1.

With the Table 1, the effective model Hamiltonian of BaAuBi can be easily expressed as: 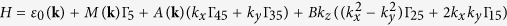, where 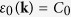

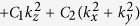
, 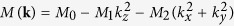, 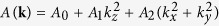.

### Effective Hamiltonian for BaAgBi

For BaAgBi, the conduction bands are Ag-5*s* states, while the valence bands are Bi-6*p* states, thus the four basis become 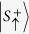, 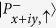, 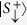 and 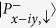. We list the character table of Γ matrices and the function *d*(**k**) (expanded as polynomials of the momentum *k*) and their transformation under time reversal in Table 2. Based on Table 2, one can get the effective model Hamiltonian for BaAgBi: 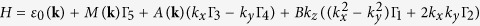, where 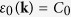

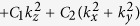
, 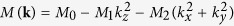, 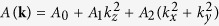.

### Band representation

At the Γ point of BZ, each state should belong to an irreducible representation of the double group of 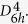 Again, applying the projection operator onto the conduction and valence states of BaAuBi, we find that 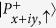 and 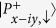 belong to representation 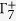, while 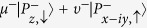 and 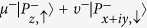 belong to representation 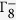, which had been marked in Fig. 2(a). Different from Γ point, the symmetry of Γ − *A* line is 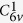. We show the compatibility relations between the double group of 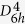 and 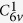 in Table 3. It is clear that the representation 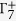 and 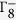, evolute to Δ_7_ and Δ_8_, respectively.

For BaAgBi, the valence/conduction states at the Γ point of BZ belong 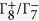, and will change to Δ_8_ and Δ_7_ along Γ − *A* line according to Table 3.

## Additional Information

**How to cite this article**: Du, Y. *et al*. Dirac and Weyl Semimetal in *XY*Bi (*X*=Ba, Eu; *Y*=Cu, Ag and Au). *Sci. Rep*. **5**, 14423; doi: 10.1038/srep14423 (2015).

## Figures and Tables

**Table 1:** The character table of Dirac Γ matrices and the polynomials of the momentum *k* for BaAuBi.

Γ matrices	representation	T
Γ_0_, Γ_5_	*R*_1_	+
{Γ_1_, Γ_2_}	*R*_14_	+
{Γ_3_, Γ_4_}	*R*_15_	+
Γ_12_, Γ_34_	*R*_2_	−
Γ_14_ + Γ_23_	*R*_3_	−
Γ_13_ − Γ_24_	*R*_4_	−
{Γ_13_ + Γ_24_, Γ_14_ − Γ_23_}	*R*_6_	−
{Γ_15_, Γ_25_}	*R*_14_	−
{Γ_35_, Γ_45_}	*R*_15_	−
*d*(*k*)	**representation**	**T**
*C*, 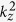 , 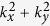	*R*_1_	+
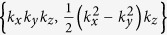	*R*_14_	−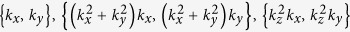	*R*_15_	−
*k*_*z*_, 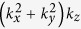 , 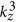	*R*_11_	−
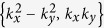	*R*	+
{*k*_*x*_*k*_*z*_, *k*_*y*_*k*_*z*_}	*R*_6_	+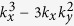	*R*	−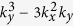	*R*	−

**Table 2:** The character table of Dirac matrices and the function *d*(*k*) of BaAgBi.

Γ matrices	representation	T
Γ_0_, Γ_5_	*R*_1_	+
{Γ_1_, Γ_2_}	*R*_14_	−
{Γ_3_, Γ_4_}	*R*_15_	−
Γ_12_, Γ_34_	*R*_2_	−
Γ_14_ − Γ_23_	*R*_3_	−
Γ_13_ + Γ_24_	*R*_4_	−
{Γ_13_ − Γ_24_, Γ_14_ + Γ_23_}	*R*_6_	−
{Γ_15_, Γ_25_}	*R*_14_	+
{Γ_35_, Γ_45_}	*R*_15_	+
***d***(***k***)	**representation**	**T**
*C*, 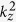 , 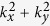	*R*	+
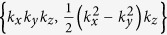	*R*_14_	−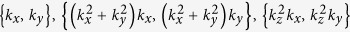	*R*_15_	−
*k*_*z*_, 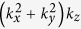 , 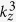	*R*	−
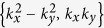	*R*_5_	+
{*k*_*x*_*k*_*z*_, *k*_*y*_*k*_*z*_}	*R*_6_	+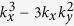	*R*_12_	−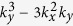	*R*	−

**Table 3:** The compatibility relations between the double group of 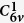 and 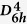 .

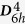	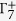	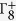	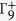	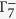	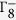	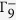
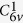	Δ_7_	Δ_8_	Δ_9_	Δ_7_	Δ_8_	Δ_9_
